# Erythema multiforme due to antitubercular drugs

**DOI:** 10.4103/0970-2113.76314

**Published:** 2011

**Authors:** Sameer Gulati, H. P. Paljor, Rohit Mahajan, Pankaj Goel

**Affiliations:** *Department of Medicine, St Stephen’s Hospital, Tis Hazari, Delhi, India E-mail: drsameergulati@gmail.com*

**Figure 1:**
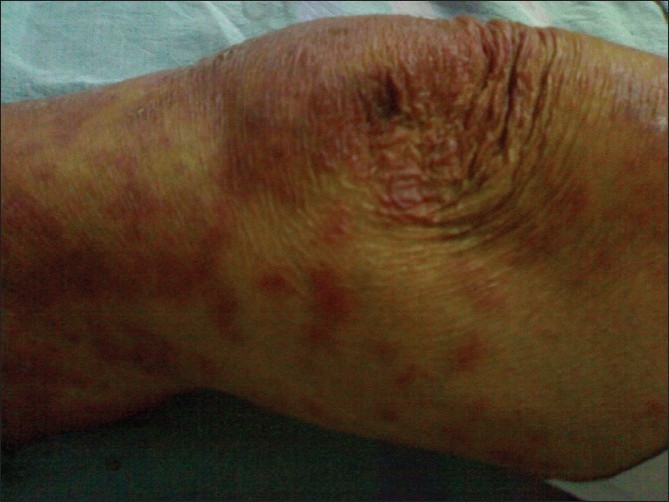
Clinical photograph showing maculopapular rashes over extremeties

**Figure 2:**
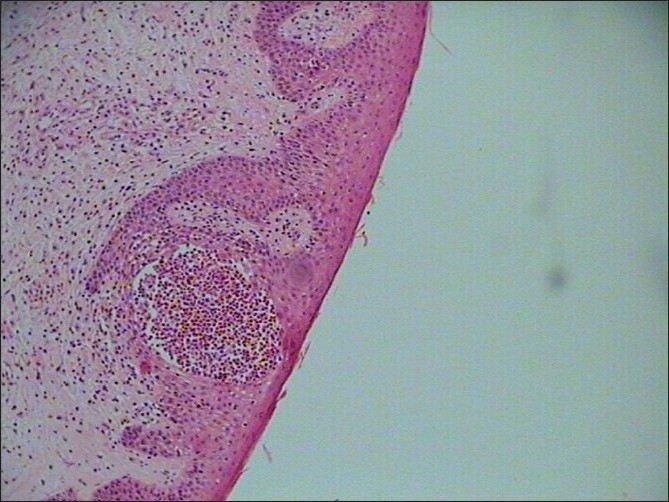
Light microscopy showing an inflammatory infilterate, keratinocyte necrosis in epidermis and dermal eosinophils

Sir,

An 84-year-old male, resident of Delhi, India presented to our hospital with complaints of maculopapular rashes in bilateral extremities since 2 days. There was no orogenital involvement. Rashes did not involve the trunk or abdomen and were also not associated with itching. There was history of starting category I antitubercular therapy, comprising of rifampicin, isoniazid, ethambutol, and pyrazinamide, two months prior to present admission for pulmonary tuberculosis. Patient was not taking any other drugs, besides antitubercular drugs. Apart from the maculopapular rashes in the extremities [Figure 1] there were no significant general or systemic examination findings. The hematological parameters revealed eosinophilia with an absolute eosinophil count of 1890/cu mm. Other biochemical parameters, including liver function tests (total bilirubin: 0.6 mg/dl, direct bilirubin: 0.1 mg/dl, serum alkaline phosphatase: 74 IU/l, serum aspartate aminotransferase: 19 IU/l, serum alanine aminotransferase: 20 IU/l) were normal. ANA and HIV Elisa were also negative. The chest X-ray done on the day of admission showed resolution as compared to previous X-rays. Skin biopsy was suggestive of erythema multiforme[Figure 2]. All the antitubercular drugs were discontinued simultaneously, and the lesions improved gradually. Skin lesions were treated conservatively with oral antihistaminics. After the lesions improved, individual antitubercular drugs were reintroduced one-by-one. The doses of the drugs were increased gradually until the maximum dose was achieved. The offending drug is usually identified during the course of this rechallenge. The drug which causes repeat skin lesions is discontinued immediately and is not given to patient again. In our patient, we could successfully reintroduce rifampicin, isoniazid, and ethambutol without any untoward skin reactions. Pyrazinamide was not given as patient had already completed 2 months of antitubercular therapy with improvement in chest X-ray. Thus, we can indirectly conclude that probably pyrazinamide was the offending drug.

Skin involvement in tuberculosis can be due to tuberculosis itself or due to the antitubercular drugs. A tuberculous chancre, erythema nodosum, scrofuloderma, lupus vulgaris, tuberculosis verrucosa cutis or a tubercular gumma are various types of cutaneous tuberculosis. Other than this, tuberculids are skin reactions that exhibit tuberculoid features histologically but do not contain detectable mycobacteria. Papulonecrotic and lichen scrofulosorum are the two types of tuberculids.

Erythema multiforme (EM) is a relatively common, acute, often recurrent inflammatory disease. Many factors have been implicated in the etiology of EM, including numerous infectious agents, drugs, physical agents, X-ray therapy, pregnancy, and internal malignancies. The drugs implicated in etiology of EM are Sulfonamides including hypoglycemics, nonsteroidal anti-inflammatory drugs (NSAIDs), anticonvulsants, barbiturates, antituberculous drugs, antibiotics, pyrazolones, phenylbutazone, oxyphenbutazone, and phenazone and salicylates. EM is commonly associated with a preceding acute upper respiratory tract infection, herpes simplex infection (HSV), or mycoplasma pneumoniae infection such as primary atypical pneumonia.[1] Studies suggest that immune complex formation and subsequent deposition in the cutaneous microvasculature may play a role in the pathogenesis of EM. A new classification, based on the pattern and distribution of cutaneous lesions, separates erythema multiforme major from Stevens–Johnson syndrome (SJS) and toxic epidermal necrolysis.[2–4] Target lesions and papules are the most characteristic eruptions. Dusky red, round maculopapules appear suddenly in a symmetric pattern on the backs of the hands and feet and the extensor aspect of the forearms and legs. The trunk may be involved in more severe cases. The diagnosis may not be suspected until the nonspecific early lesions evolve into target lesions during a 24- to 48-h period. The classic “iris” or target lesion results from centrifugal spread of the red maculopapule to a circumference of 1–3 cm as the center becomes cyanotic, purpuric, or vesicular. The mature target lesion consists of two distinct zones: an inner zone of acute epidermal injury with necrosis or blisters and an outer zone of erythema. There may be a middle zone of pale edema. Mild cases are not treated. Patients with many target lesions respond rapidly to a 1- to 3-week course of prednisone. Oral acyclovir (400 mg twice a day) used continually prevents herpes-associated recurrent EM in many cases. If these treatments fail, dapsone or antimalarial drugs may be tried. Azathioprine was used successfully in patients with severe disease for whom all other treatments had failed.
